# Ingested PET microplastics alter the metabolomic profile of the porcine pancreas

**DOI:** 10.1038/s41598-025-21915-5

**Published:** 2025-11-10

**Authors:** Karol Mierzejewski, Aleksandra Kurzyńska, Monika Golubska, Ismena Gałęcka, Jarosław Całka, Iwona Bogacka

**Affiliations:** 1 Department of Animal Anatomy and Physiology, University of Warmia and Mazury in Olsztyn, Oczapowskiego 1a, Olsztyn, 10-719 Poland; 2 Department of Epizootiology, University of Warmia and Mazury in Olsztyn, Oczapowskiego 14, Olsztyn, 10-719 Poland; 3 Department of Clinical Physiology, University of Warmia and Mazury in Olsztyn, Oczapowskiego 14, Olsztyn, 10-719 Poland

**Keywords:** Pancreas, Diabetes, Insulin resistance, Glucose, Microplastics, Diabetes, Metabolism

## Abstract

Microplastics and their effects on health are a growing concern. While their full impact is not yet known, they are not harmless. Pancreatic diseases are increasingly common, even in children, which was once rare. This study examined pancreatic changes caused by PET microplastics in young organisms. Gilts received low (0.1 g/day) or high (1 g/day) doses of PET microplastics for four weeks, and their pancreases were analyzed using UPLC-MS/MS. Blood insulin levels and other biochemical parameters were also measured. PET microplastics altered physiological processes in the pancreas, increasing glucose, γ-aminobutyric acid, lysophosphatidylcholine, and lysophosphatidylethanolamine levels in tissues. They also elevated blood insulin concentrations and affected in a dose-dependent manner lipase, cholesterol, and calcium levels. These findings suggest that PET microplastics may contribute to insulin resistance and pancreatitis.

**Supplementary Information:**

The online version contains supplementary material available at 10.1038/s41598-025-21915-5.

## Introduction

Microplastics (MPs), defined as plastic particles smaller than 5 mm, have become ubiquitous in the environment due to widespread plastic production and inadequate waste management^1^. The pervasive presence of microplastics in ecosystems, food and beverages raises major concerns about the potential impact on human health, particularly through ingestion and subsequent accumulation in various organs. The accumulation of MPs has already been demonstrated in many tissues including the brain, liver, placenta, testis, and blood^2–4^. Plastic particles have been shown to induce neurotoxicity, impair the synthesis of steroid hormones, impact the quality of oocytes and sperm, promote cardiovascular disease, develop microbial dysbiosis as well as hepatic inflammation^5^. There is also growing body of evidence that microplastics impair the pancreatic function^6^.

The pancreas is a vital organ that fulfils both endocrine and exocrine functions that are essential for the proper functioning of the organism. The central role of the pancreas is to regulate macronutrient digestion and thus metabolism and energy homeostasis through the release of various digestive enzymes and pancreatic hormones^7^. It consists of two structurally and functionally integrated glandular systems, namely the exocrine and endocrine pancreas. The largest part of this organ consists of acinar or exocrine cells that secrete pancreatic juice, which contains digestive enzymes such as amylase, pancreatic lipase and trypsinogen, into the pancreatic duct and accessory pancreatic duct^8^. The endocrine pancreas, on the other hand, consists of the islets of Langerhans, which secrete hormones such as insulin directly into the bloodstream to regulate blood glucose levels^9^. Dysfunction of the pancreas can lead to serious diseases such as insulin resistance, diabetes, chronic pancreatitis and pancreatic cancer.

There is evidence that polystyrene (PS) MPs exacerbate pancreatic injury and inflammation in acute pancreatitis in mice^6^. In addition, exposure to PS MPs induces insulin resistance in mice, mediated through regulating the gut microbiota, stimulating inflammation and inhibiting the insulin signaling pathway^10^. Another study showed that MPs induce oxidative stress and activate the GRP78/CHOP/Bcl-2 signaling pathway to increase pancreatic apoptosis in mice^11^. In our previous study, we found that PET MPs in piglets alter the expression of miRNA in serum-derived extracellular vesicles (EVs), which are associated with insulin resistance, type II diabetes and the development of pancreatic cancer^12^. The available literature on the effects of microplastics on the pancreas is patchy and a comprehensive understanding of the mechanisms is still lacking. Therefore, the aim of this study was to investigate the effects of oral exposure to PET microplastics on the global metabolomic profile of the pancreas in piglets using UPLC-MS/MS analysis. In addition, insulin levels and various biochemical parameters in the blood were assessed.

The selection of PET microplastics for this study was justified by their widespread use in consumer products such as beverage bottles, food packaging, and textiles, which makes them a major source of human exposure^13,14^. PET particles have been detected in human blood and feces, indicating that gastrointestinal absorption and systemic distribution occur under real-life exposure conditions^15–17^. Furthermore, immature organisms – the subject of this study – are particularly vulnerable to environmental toxicants, and existing data suggest that daily PET exposure in infants is significantly higher than in adults^18^. Thus, PET represents a biologically and environmentally relevant microplastics for evaluating early-life effects on sensitive organs such as the pancreas.

## Results

### Effect of a low dose of PET microplastics on global metabolomic profile of the pancreas

**Fig. 1:**
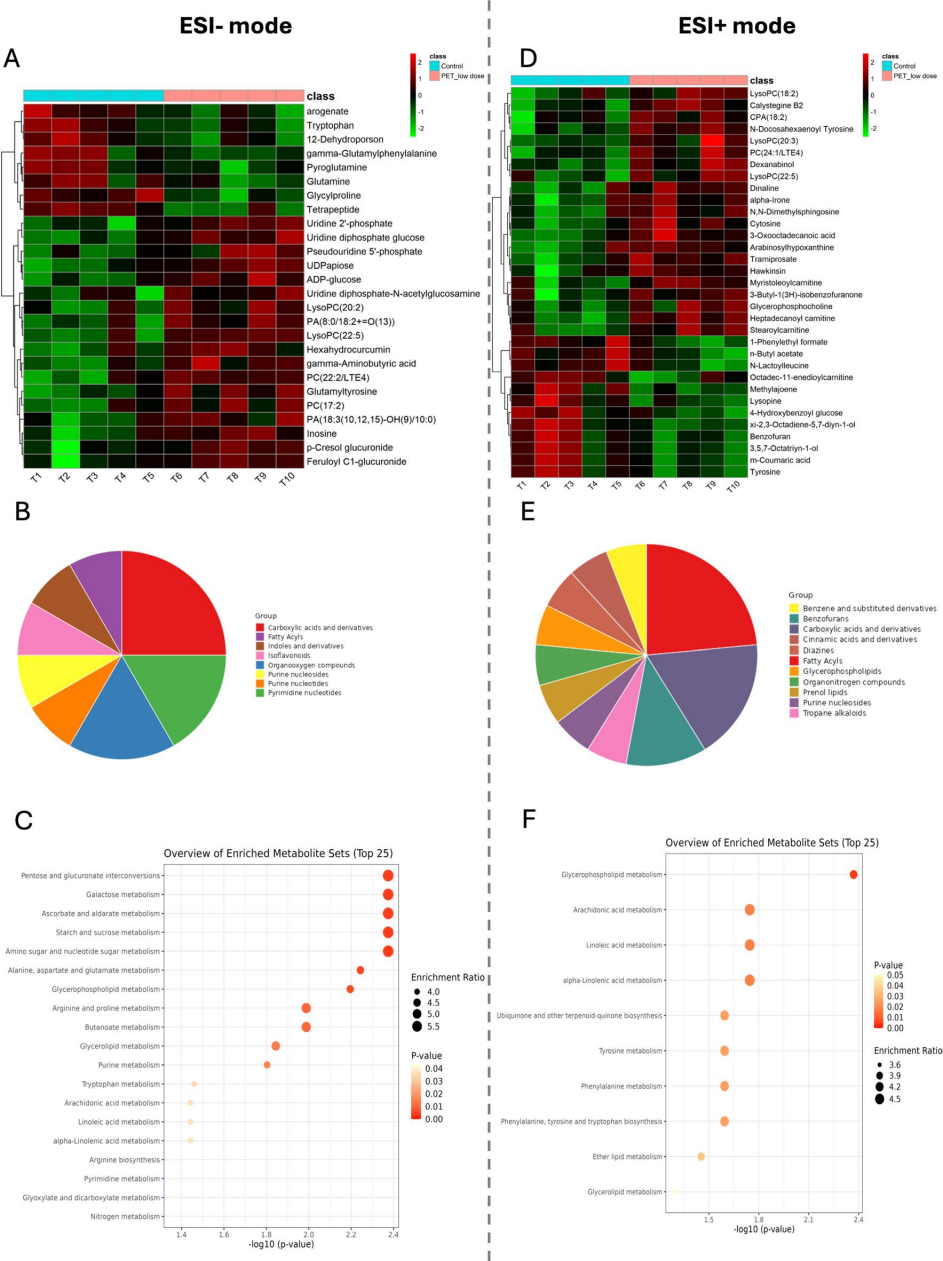
The analysis of untargeted metabolome profiles of the pancreas isolated from the piglets treated with a low (LD) of PET microplastics compared to the control group in negative ionization mode (ESI-) or positive ionization mode (ESI+). (**A**) Hierarchical cluster analysis of biomarkers under the influence of LD of PET microplastics identified in ESI- mode. Color intensity correlates with degree of increase (red) and decrease (green) relative to the mean metabolite ratio. (**B**) Distribution of differentially regulated metabolites by different metabolite classes identified in ESI- mode under the influence of LD of PET microplastics. (**C**) Dot plot of network analysis in differentially regulated metabolites identified in ESI- mode according to KEEG and MetaboAnalyst under the influence of LD of PET microplastics. (**D**) Hierarchical cluster analysis of biomarkers under the influence of LD of PET microplastics identified in ESI + mode. Color intensity correlates with degree of increase (red) and decrease (green) relative to the mean metabolite ratio. (**E**) Distribution of differentially regulated metabolites by different metabolite classes identified in ESI + mode under the influence of LD of PET microplastics. (**F**) Dot plot of network analysis in differentially regulated metabolites identified in ESI + mode according to KEGG and MetaboAnalyst under the influence of LD of PET microplastics.

In the ESI- mode, our analysis revealed 26 biomarkers – 8 of them were downregulated, while 18 were upregulated (Fig. 1A). Most of these differentially regulated metabolites belong to the following classes: carboxylic acids and derivatives (gamma-aminobutyric acid; gamma-glutamylphenylalanine; glutamine; glycylproline; glutamyltyrosine; pyroglutamine), organooxygen compounds (pseudouridine 5’-phosphate; uridine 2’-phosphate; p-cresol glucuronide) or pyrimidine nucleotides (uridine diphosphate glucose; uridine diphosphate-N-acetylglucosamine; UDP-D-apiose) (Fig. 1B). According to the Kyoto Encyclopedia of Genes and Genomes (KEGG) analysis, the differentially regulated metabolites are involved in processes such as pentose and glucuronate interconversions, galactose metabolism, starch and sucrose metabolism, glycerolipid metabolism or arachidonic acid metabolism (Fig. 1C). Detailed results of the normalized data, HCA and KEGG analysis are presented in the Supplemental Materials (Table S1 a–c).

In ESI + mode, the analysis revealed 33 biomarkers – 12 of them was downregulated, while 21 were upregulated (Fig. 1D). Most of these differentially regulated metabolites belong to the following classes: fatty acyls (stearoylcarnitine; heptadecanoyl carnitine; CPA(18:2(9Z,12Z)/0:0); 3-oxooctadecanoic acid; 3,5,7-octatriyn-1-ol; xi-2,3-octadiene-5,7-diyn-1-ol; myristoleoylcarnitine); amino acids, peptides, and analogues (N-lactoylleucine, lysopine, hawkinsin), carboxylic acids and derivatives (L-tyrosine; hawkinsin; n-butyl acetate; L-lysopine; N-lactoylleucineor) or benzofurans ((S)-3-butyl-1(3 H)-isobenzofuranone; benzofuran) (Fig. 1E). According to the KEGG analysis, the differentially regulated metabolites are involved in processes such as glycerophospholipid metabolism, arachidonic acid metabolism, linoleic acid metabolism or glycerolipid metabolism (Fig. 1F). Detailed results of the normalized data, HCA and KEGG analysis are presented in the Supplemental Materials (Table S2a–c).

### Effect of a high dose of PET microplastics on global metabolomic profile of the pancreas

**Fig. 2:**
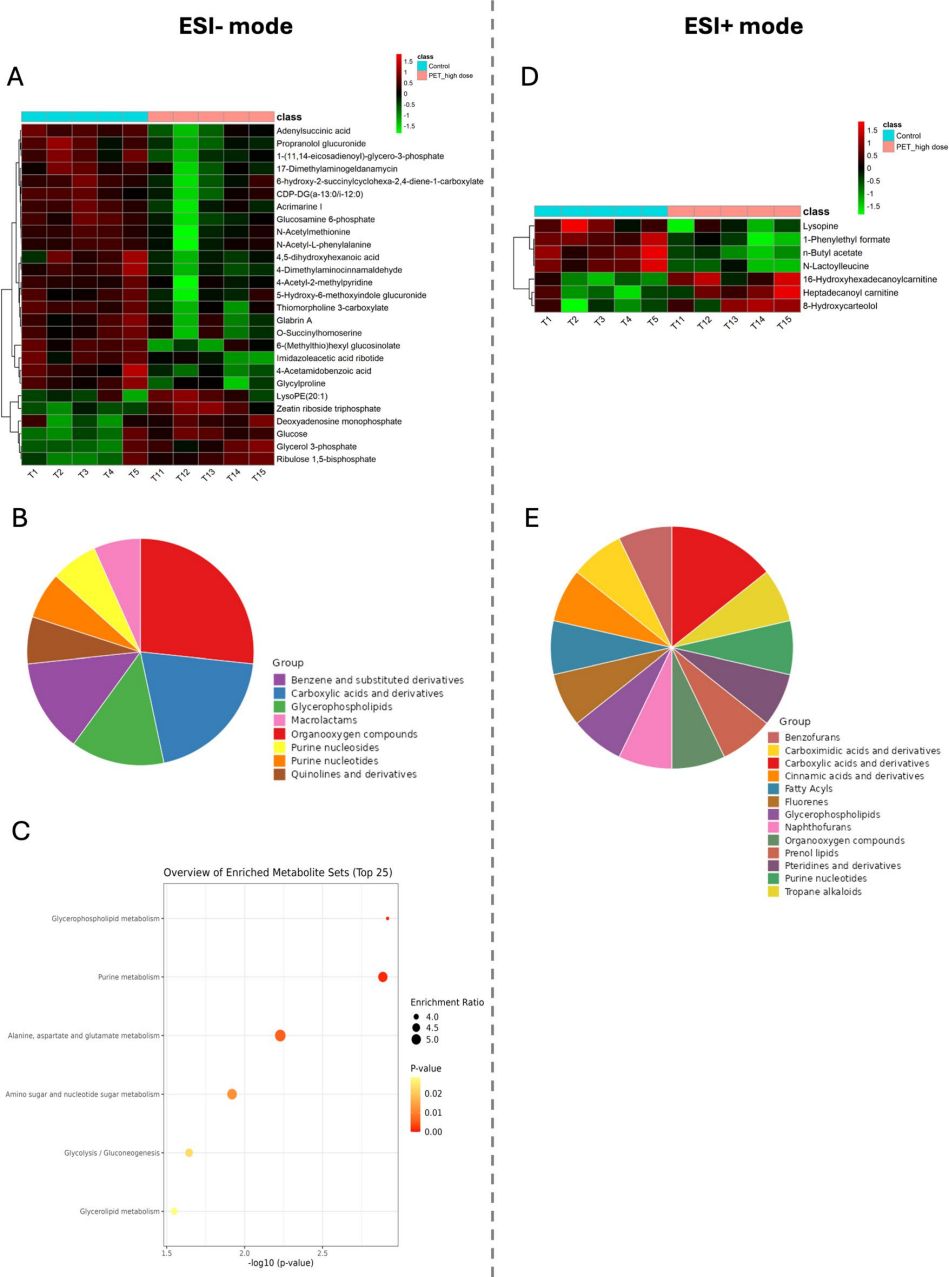
The analysis of untargeted metabolome profiles of the pancreas isolated from the piglets treated with a high dose (HD) of PET microplastics compared to the control group in negative ionization mode (ESI-) or positive ionization mode (ESI+). (**A**) Hierarchical cluster analysis of biomarkers under the influence of HD of PET microplastics identified in ESI- mode. Color intensity correlates with degree of increase (red) and decrease (green) relative to the mean metabolite ratio. (**B**) Distribution of differentially regulated metabolites by different metabolite classes identified in ESI- mode under the influence of HD of PET microplastics. (**C**) Dot plot of network analysis in differentially regulated metabolites identified in ESI- mode according to KEGG and MetaboAnalyst under the influence of HD of PET microplastics. (**D**) Hierarchical cluster analysis of biomarkers under the influence of HD of PET microplastics identified in ESI + mode. Color intensity correlates with degree of increase (red) and decrease (green) relative to the mean metabolite ratio. (**E**) Distribution of differentially regulated metabolites by different metabolite classes identified in ESI + mode under the influence of HD of PET microplastics.

In the ESI- mode, we identified 27 biomarkers – 21 of them were downregulated, while 6 were upregulated (Fig. 2A). Most of these differentially regulated metabolites belong to the following classes: organooxygen compounds (D-glucose; glucosamine 6-phosphate; 5-hydroxy-6-methoxyindole glucuronide; 6-(methylthio)hexyl glucosinolate; 4-acetyl-2-methylpyridine), carboxylic acids and derivatives (N-acetyl-L-phenylalanine; glycylproline; N-acetyl-L-methionine; thiomorpholine 3-carboxylate) or glycerophospholipids (glycerol 3-phosphate; 1-(11Z,14Z-eicosadienoyl)-glycero-3-phosphate) (Fig. 2B). According to the KEGG analysis, the differentially regulated metabolites are involved in processes such as glycerophospholipid metabolism, purine metabolism, alanine, aspartate and glutamate metabolism, amino sugar and nucleotide sugar metabolism, glycolysis/gluconeogenesis and glycerolipid metabolism (Fig. 2C). Detailed results of the normalized data, HCA and KEGG analysis are presented in the Supplemental Materials (Table S3a–c).

In ESI + mode, the analysis revealed 7 biomarkers – 4 of them was downregulated, while 3 were upregulated (Fig. 2D). The differentially regulated metabolites belong to the following classes: carboxylic acids and derivatives (creatinine; L-glutamic gamma-semialdehyde; proline betaine), pteridines and derivatives (riboflavin) or fatty acyls (5-oxo-12-HETE) (Fig. 2E). These differentially regulated metabolites were not assigned to any KEGG pathway. Detailed results of the normalized data, HCA and KEGG analysis are presented in the Supplemental Materials (Table S4a,b).

### Effect of PET microplastics on insulin and blood biochemical parameters

**Fig. 3:**
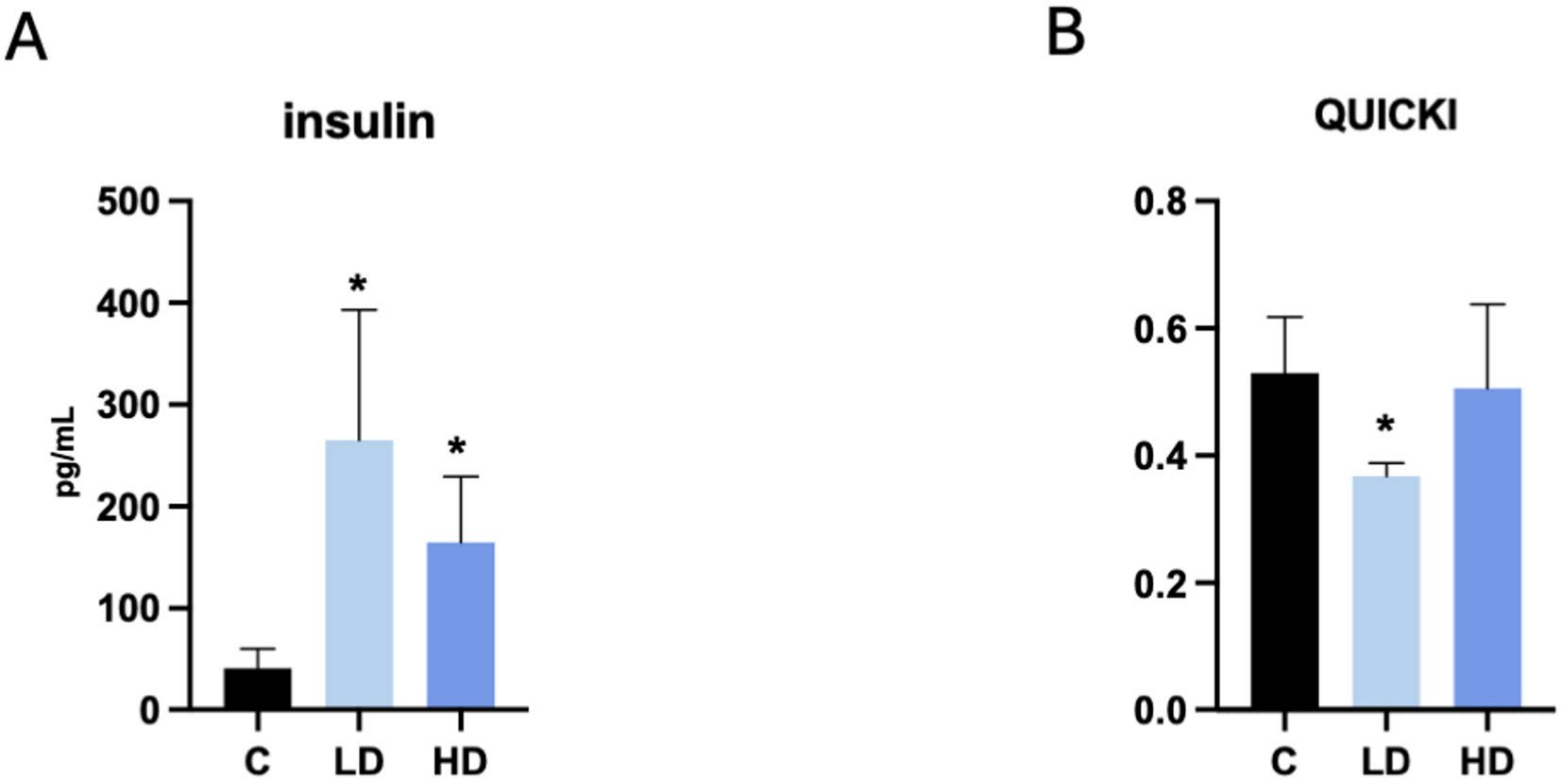
Blood (**A**) insulin levels and (**B**) QUICKI index in piglets treated with a low (LD) or high (HD) dose of PET microplastics, compared to a control group (C).

**Fig. 4:**
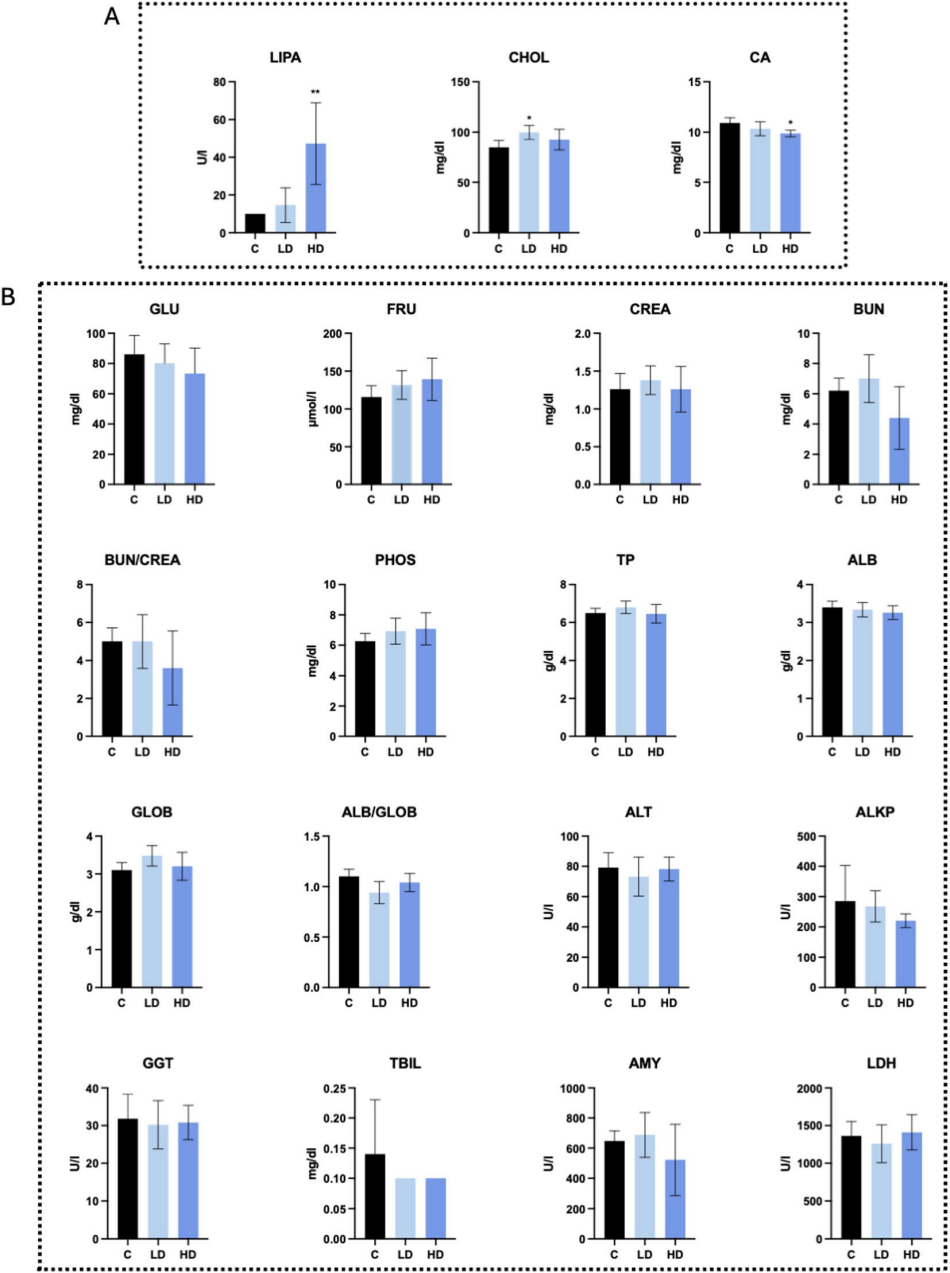
Biochemical parameters measured in the blood of piglets treated with a low (LD) or high (HD) dose of PET microplastics, compared to a control group (C). Panel (**A**) presents significant differences in serum concentrations of lipase (LIPA), cholesterol (CHOL), and calcium (CA). (**B**) Comprehensive serum biochemical analysis across groups. No significant differences were observed among groups for glucose (GLU), fructosamine (FRU), creatinine (CREA), blood urea nitrogen (BUN), BUN/CREA ratio, phosphorus (PHOS), total protein (TP), albumin (ALB), globulin (GLOB), albumin/globulin ratio (ALB/GLOB), alanine aminotransferase (ALT), alkaline phosphatase (ALP), gamma-glutamyl transferase (GGT), total bilirubin (TBIL), amylase (AMY), or lactate dehydrogenase (LDH). Data are presented as mean ± SD.

Serum insulin concentrations were higher in piglets treated with either a low or high dose of PET microplastics than in control subjects (Fig. 3A). The quantitative insulin sensitivity check index (QUICKI) was significantly reduced in the presence of a low dose, while it did not change after a high dose of PET microplastics (Fig. 3B). In addition, serum cholesterol levels (CHOL) increased after treatment with a low dose of PET microplastics, while a high dose of PET increased pancreatic lipase (LIPA) levels and decreased calcium (Ca) levels (Fig. 4A). Other biochemical parameters in serum were not affected by PET microplastics (Fig. 4B).

## Discussion

Pancreatic diseases are an increasing concern in highly developed countries^19^. Similarly, microplastics have become a significant global problem, as there is growing evidence that they contribute to various disorders. Therefore, we investigated the effects of PET microplastics on the pancreatic metabolome to better understand their potential role in exacerbating pancreatic diseases. In this study, we demonstrated that PET microplastics alter pancreatic metabolite profiles and that dysregulation is associated with insulin resistance, glucose and lipid homeostasis, and oxidative stress.

One of the most important functions of the pancreas is to regulate glucose homeostasis by releasing glucagon from α-cells during hypoglycemia and insulin from β-cells when glucose levels rise^8^. It has been frequently reported that prolonged hyperglycemia impairs pancreatic physiology. For example, elevated glucose shifts the balance of BCL family proteins toward apoptosis, which promotes β-cell loss^20^, and renders β-cells hypersensitive to glucose upon chronic in vitro exposure, thereby lowering the threshold for insulin release^21–23^. High glucose can also activate pancreatic stellate cells via the PKC-p38 MAPK pathway, upregulate α-SMA and collagen I, and thus promote fibrotic remodeling^24^.

Our metabolomics data indicate that both doses of PET microplastics may disrupt key pathways of glucose metabolism in the pancreas. Once glucose is transported into the cytosol via the GLUT, it is immediately phosphorylated by hexokinase or glucokinase to glucose-6-phosphate (G6P), preventing its efflux and directing it into downstream metabolic pathways^25^. In piglets treated with a high dose of PET microplastics, we observed a marked accumulation of free glucose and glycerol-3-phosphate, a glycolytic intermediate and a precursor for triglyceride synthesis^26^, suggesting impairment of the later steps of glycolysis. A concurrent increase in ribulose-1,5-bisphosphate indicates activation of the pentose phosphate pathway, a typical response to an increased NADPH requirement for ROS detoxification^27^. In addition, increased G6P levels indicate upregulation of the hexosamine biosynthetic pathway, leading to excessive O-GlcNAcylation of regulatory proteins and impaired insulin signaling in β-cells^28^. In contrast, a low dose of PET microplastics significantly increased the level of UDP-glucose, an active donor for glycogen synthesis and protein glycosylation, suggesting a shift of the G6P pool towards glucose storage and protein modification^29^. At the same time, the increased UDP-GlcNAc level confirms the activation of the hexosamine biosynthetic pathway, which promotes O-GlcNAcylation of key regulatory proteins and increases the susceptibility of β-cells to dysfunctional insulin signaling^30^.

Our study showed a dose-dependent effect of PET microplastics on glucose metabolism in the pancreas. Under the influence of a low dose of PET microplastics, the cells appear to be able to buffer excess glucose through glycogen synthesis and O-GlcNAcylation. In contrast, a high dose seems to block the major glycolytic flux, leading to accumulation of glucose and G6P in pancreatic tissue and activation of alternative pathways of glucose utilization, such as the pentose phosphate and hexosamine biosynthetic pathways. Interestingly, despite these local metabolic changes, blood glucose levels remained unchanged, suggesting that the high dose of PET microplastics leads to local storage of glucose in pancreatic cells rather than systemic hyperglycemia. Taken together, these results suggest that the increase in pancreatic glucose levels induced by PET microplastics may contribute to β-cell failure, insulin resistance, and fibrotic changes characteristic of type 2 diabetes. However, further studies are needed to determine the mechanism behind this effect, with particular attention to the role of microplastics in modulating glucose transporter activity.

Our study showed that both doses of PET microplastics increase serum insulin levels. In healthy individuals, insulin binds to cell surface receptors and triggers responses that enable the uptake of glucose from the blood. In the case of insulin resistance, the cells respond inadequately to insulin, elevating glucose absorption and raising blood glucose levels. To compensate for this, the pancreas increases insulin production, leading to hyperinsulinemia. Impaired insulin secretion in metabolic disorders can precede and worsen insulin resistance^31^. Our analysis revealed that the QUICKI was significantly lower in piglets treated with a low dose of PET microplastics, suggesting an increased risk of insulin resistance. Our results are consistent with reports from other groups. There is evidence that PS microplastics increase the risk of insulin resistance in mice by affecting the gut-liver axis^32^. Another study in mice showed insulin resistance after PS exposure in association with increased plasma proinflammatory cytokines such as tumor necrosis factor-α and interleukin-1β^10^. Polystyrene microplastics also elevated ROS in the liver, which disrupted the PI3K/Akt signaling pathway and led to insulin resistance^33,34^. It is worth noting that our previous study demonstrated the effects of PET microplastics on the expression of porcine serum-derived extracellular vesicles miRNA. These differentially regulated miRNAs can be involved in the development of metabolic syndrome, insulin resistance and type 2 diabetes^12^. There is evidence that insulin resistance is associated with a high rate of cholesterol synthesis and low cholesterol absorption^35^. Interestingly, our study showed a stimulatory effect of a low dose of PET microplastics on serum cholesterol levels, supporting the hypothesis that PET microplastics may significantly disrupt insulin homeostasis. It should be emphasized that the observed hyperinsulinemia and altered glucose metabolism indicate that PET microplastics could play a role in the pathophysiology of metabolic syndrome, in addition to known risk factors such as obesity and physical inactivity^36^.

An important element of our research was the finding of a stimulatory effect of a low dose of PET microplastics on γ-aminobutyric acid (GABA) levels in the pancreas. It is known that pancreatic β-cells synthesize GABA from glutamic acid by GAD and store it in the synaptic macrovesicles with insulin granules until the time of secretion^37^. GABA plays a fundamental role in the regulation of pancreatic β-cells by suppressing and stimulating glucagon and insulin secretion, respectively. In addition, GABA can regenerate β-cells after destruction^38^. This phenomenon could be a compensatory mechanism of the pancreas in response to the increased glucose levels in pancreatic cells under the influence of microplastics. However, the significance of this mechanism requires clarification in subsequent studies.

An important group of metabolites affected by PET microplastics in the pancreas are lysophospholipids such as lysophosphatidylcholine (lysoPC, LPC) and lysophosphatidylethanolamine (lysoPE, LPE). They are bioactive lipids with diverse physiological and pathological functions in the pancreas. LPC is known as an endogenous mediator that triggers insulin secretion in pancreatic β-cells. LPC has been reported to increase the transcriptional activity of NF-kB and AP-1 and induce apoptosis by activating extracellular signal-regulated kinase (ERK) 1/2, c-Jun NH2-terminal kinase/stress-activated protein kinase (JNK/SAPK) and p38 MAP kinases in rat pancreatic AR42J cells^39^. Moreover, the higher LPC and LPE levels were associated with the risk of pancreatic ductal carcinoma^40^. The present study showed that a low dose of PET microplastics upregulated the levels of LPC (18:2), LPC (20:3), LPC (20:2) and LPC (22:5) in the pancreas. In turn, a high dose of PET microplastics increased LPE (20:1) in the pancreas. The results suggest that exposure to PET microplastics, even at low doses, can significantly alter lipid metabolism in the pancreas, potentially increasing the risk of pancreatic diseases, including pancreatitis, or the risk of pancreatic cancer. Another metabolite that may serve as a marker for chronic pancreatitis is glycerophosphocholine (GPC), which is catalyzed from LPC^41^. Numerous studies show elevated phosphocholine and GPC levels as hallmarks of cancer^42^. We observed increased pancreatic GPC following low dose PET exposure.

We found that PET microplastics increased the level of pancreatic lipase in serum fourfold compared to the control. The measurement of pancreatic lipase is routinely used in clinical practice. Their elevated levels correlate strongly with various pathological conditions, including acute, and chronic pancreatitis^43^. Moreover, there is evidence that persistently elevated or increasing combined levels of amylase and lipase in serum are reliable indicators of pancreatic injury^44^. Although our studies showed no significant change in serum amylase levels due to PET exposure, given the significantly elevated lipase levels and the literature data indicating that microplastics cause pancreatic injury in mice^6^, we suggest that future research should consider assessing the extent of tissue damage over time and the levels of pancreatic enzymes in serum. Furthermore, our study revealed that high dose of PET microplastics also decrease serum calcium levels. It is well documented that abnormal regulation of Ca^2+^ signaling is one of the central triggers for the pathogenesis of acute pancreatitis^45^. Hypocalcemia also was significantly more frequent in patients with a severe form of acute pancreatitis than in patients with a mild form^45^.

It should also be emphasized that the untargeted metabolomics approach, while offering broad analytical coverage, has certain limitations. Metabolite identification was primarily based on matches with public databases, which in some cases may result in putative rather than definitive compound annotations. Additionally, this technique can be sensitive to matrix effects and ionization variability between compounds. However, to mitigate these issues and ensure the reliability of our data, we applied several quality control measures, including analysis in both ESI + and ESI- modes, the use of pooled QC samples, data normalization, and stringent statistical thresholds^46,47^.

In summary, we demonstrated for the first time that PET microplastics affect the metabolomic profile of the pancreas. Specifically, PET microplastics lead to alterations in glucose metabolism, cause early hyperinsulinemia, and induce changes in lipid metabolism, while also perturbing blood biochemical parameters (serum insulin, lipase, cholesterol and calcium). These disturbances may represent the early stages of β-cell dysfunction and contribute to inflammation, β-cell apoptosis and fibrosis – key features of insulin resistance, type 2 diabetes and chronic pancreatitis. However, to confirm the underlying mechanisms and assess the long-term consequences, further functional studies are required.

## Materials and methods

### Animals

All experimental protocols were approved by the Local Ethics Committee of the University of Warmia and Mazury in Olsztyn (Decision No. 10/2020, dated February 26, 2020). All methods were carried out in accordance with Polish law, which defines the conditions and methods of conducting experiments on animals, and the European Community Directive (EU Directive 2010/63/EU) on the ethical use of experimental animals. All methods were performed in accordance with ARRIVE guidelines. All animals (sourced from a farm in Lubawa, Poland) were housed in breeding pens under standard laboratory conditions and were provided with free access to fresh water (*ad libitum*) and an age-appropriate feed mixture. The temperature in the animal pens was maintained at 20–22 °C and the humidity was between 55% and 60%. All plastic objects were removed from the animals’ environment. The watering through and feeders in the experimental pens were made of stainless steel. The equipment on the farm where the gilts were reared before the experiment was also made of stainless steel. The elements of the environment that guaranteed a high level of welfare were made of wood or bedding. The experiment lasted 4 weeks and was carried out on 8-week-old (Pietrain x Duroc) immature gilts (*n* = 15) with an estimated body weight of 20 kg. The animals were divided into three groups: (1) control group (C; *n* = 5), which received empty gelatine capsules *per os*; (2) experimental group (LD; *n* = 5), which received a low dose of PET MPs (0.1 g/pig/day in gelatine capsules) *per os*; (3) experimental group (HD; *n* = 5), which received a high dose of PET MPs (1 g/pig/day in gelatine capsules) *per os*. The gelatine capsules were administered to the gilts 1 h before morning feeding. The polyethylene terephthalate (Cat. No. ES306031/1, Goodfellow Cambridge Ltd., England) was analyzed by dynamic light scattering to determine the particle size distribution and microscopic analysis was used to visualize the shape and appearance of the particles as previously described^48^. Briefly, the plastic particles ranged from 7.6 to 416.9 μm, with most particles having a diameter of 158.5 μm. Different shapes of the particles (spherical, fibrous, irregular) were observed. The PET particles had both sharp and rounded edges. In this study, we administered two doses of PET microplastics: a low dose of 0.1 g/day/animal and a high dose of 1 g/day/animal. These doses were chosen based on our previous study^49–51^ and estimates indicating that humans ingest approximately 0.1 to 5 g of microplastics per week^52^. Thus, the weekly equivalents used in the study – around 0.7 g and 7 g – represent realistic levels of human exposure within the estimated average intake range. After 4 weeks, the piglets were euthanized. Before euthanasia, blood was collected from the jugular vein. The euthanasia protocol was based on the use of atropine (0.05 mg/kg i.m., Polfa, Poland), followed by xylazine (3 mg/kg i.m., Vet-Agro, Poland) and ketamine (6 mg/kg i.m., Vetoquinol Biowet, Poland). After approximately 20 min, when the gilts were unconscious, an overdose of sodium pentobarbital (0.6 mL/kg i.v., Biowet, Poland) was applied^53^. Once it was confirmed that vital functions had ceased (absence of pupillary reflex, pulse and respiration), the body and tail of the pancreas was immediately collected for further analyses.

### Sample preparation

The pancreatic tissue samples were thawed on ice. Approximately 100 mg of each sample was weighed into a tube, to which 80% methanol (8 µL/mg of raw material) and two 5 mm metal spheres were added. All samples were ground twice for 180 s at 65 Hz, followed by sonication for 30 min at 4 °C. Then each sample was kept at -20 °C for 1 h. Afterwards, the samples were centrifuged for 15 min at 12,000 rpm at 4 °C. Finally, 200 µL of the supernatant and 5 µL of DL-o-chlorophenylalanine (0.2 mg/mL) were transferred to a vial for UPLC-MS/MS analysis. Quality control (QC) samples were used to evaluate the methodology. The same amount of extract was obtained from each sample and mixed as QC samples. The QC sample was prepared using the same sample preparation procedure^54^.

### UPLC-MS/MS

Separation of compounds was performed by ultra-performance liquid chromatography (UPLC) coupled with tandem mass spectrometry using a Q Exactive Plus MS (Thermo Fisher Scientific, Waltham, MA, United States) and screened with electrospray ionization mass spectrometry (ESI-MS). The LC system comprised an ACQUITY UPLC HSS T3 column (100 × 2.1 mm, 1.8 μm) (Waters Corporation, Milford, MA, United States). The mobile phase was composed of solvent A (0.05% formic acid in water) and solvent B (acetonitrile) with a gradient elution (0–1 min, 5% B; 1–12 min, 5%–95% B; 12–13.5 min, 95% B; 13.5–13.6 min, 95%–5% B; 13.6–16 min, 5% B). The flow rate of the mobile phase was 0.3 mL/min. The column temperature was maintained at 40 °C, and the sample manager temperature was set at 4 °C. Mass spectrometry parameters in ESI + and ESI- modes are listed as follows: for ESI + mode, the heater temperature was 300 °C, the sheath gas flow rate was 45 arbitrary units (arb), the auxiliary gas flow rate was 15 arb, the sweep gas flow rate was 1 arb, the spray voltage was 3.0 kV, the capillary temperature was 350 °C, and the S-Lens RF level was 30%. For ESI- mode, the heater temperature was 300 °C, the sheath gas flow rate was 45 arb, the auxiliary gas flow rate was 15 arb, the sweep gas flow rate was 1 arb, the spray voltage was 3.2 kV, the capillary temperature was 350 °C, and the S-Lens RF level was 60%. The mass spectrometry scan modes included Full Scan (m/z 70–1050, resolution: 70,000) and data-dependent MS2 (dd-MS2, TopN = 10, resolution: 17,500) with higher-energy collisional dissociation (HCD) as the collision mode.

### Data processing and statistical analysis

Raw LC-MS/MS data were acquired and aligned using the Compound Discoverer (v. 3.0, Thermo Fisher Scientific, Waltham, MA, United States) based on the m/z value and retention time (RT) of the ion signals. The ions from the ESI- and ESI + modes were merged and imported into SIMCA-P software (version 14.1, Sartorius, Göttingen, Germany) for multivariate analysis. The spectral data were normalized and automatically scaled prior to statistical analysis. Principal component analysis (PCA) was initially used as an unsupervised method to visualise the data and identify outliers. Supervised regression modelling was performed on the dataset using Partial Least Squares Discriminant Analysis (PLS-DA) or Orthogonal Partial Least Squares Discriminant Analysis (OPLS-DA) to identify potential biomarkers. The quality of the models was assessed using the relevant R2 and Q2 values, where R2 indicates the variance explained in the model and Q2 reflects the predictability of the model. The importance of each ion in the PLS-DA or OPLS-DA models was assessed using the VIP (Variable Importance in the Projection) score, where VIP values greater than 1.5 were considered significant. Biomarkers were filtered and confirmed by combining the results of the VIP scores and the t-test (*p* < 0.05). The chemical structures and IDs of the metabolites were identified using the Human Metabolome Database^55^, the KEGG Database^56,57^, the PubChem Compound ID Database^58^ and the ChemSpider Database (https://www.chemspider.com/Default.aspx). In the multivariate analyses, hierarchical cluster analysis (HCA) with the measured Euclidean distance and the average cluster algorithm was used to visualize the differences in the concentration of each statistically significant metabolite between the groups in two different ESI modes. Metabolites were then assigned to substance groups and pathway enrichment analysis was performed using MetaboAnalyst 5.0^59,60^.

### Biochemical analysis

Prior to blood sampling, the pigs were subjected to a 24-hour fasting period and had ad libitum access to water. Blood was collected by puncturing the external jugular vein into a tube containing a coagulation activator. It was then left to stand at room temperature for at least 30 min. To obtain the serum, the collected material was centrifuged at room temperature for 10 min at 3,000 rpm. The collected serum was aliquoted and stored at -20 °C for biochemical analyses. The biochemical analysis included the determination of the concentration of albumin (ALB), alkaline phosphatase (ALKP), alanine aminotransferase (ALT), amylase (AMY), blood urea nitrogen (BUN), total calcium, non-ionized (Ca), cholesterol (CHOL), creatinine (CREA), fructosamine (FRU), gamma-glutamyl transferase (GGT), globulins (GLOB), glucose (GLU), lactate dehydrogenase (LDH), lipase (LIPA), phosphorus (PHOS), total bilirubin (TBIL) and total protein (TP) using the Idexx Catalyst One Chemistry Analyser (IDEXX Laboratories, Inc., United States) according to the manufacturer’s instructions. Based on the results obtained, the analyser also calculated the ALB/GLB, BUN/CREA ratios and concentration of globulins. For parameters whose concentration did not exceed the minimum value detectable by the analyser (FRU 100 µmol/l, TBIL 0.1 mg/dl, LIPA 10 U/l), the minimum value of the assay (FRU 100 µmol/l, TBIL 0.1 mg/dl, LIPA 10 U/l) was assumed for further analysis. The concentration of insulin in the serum was determined using a commercially available ELISA kit (ELK Biotechnology, cat: ELK1385) according to the manufacturer’s protocol and as previously described^61^. The range of standard curves was 7.82–500 pg/mL. Absorbance values were measured at 450 nm using an Infinite M200 Pro reader with Tecan i-control software (version 1.11, Tecan, Switzerland). The intra- and inter-assay coefficients of variation of the ELISA assay for insulin were < 8% and < 10%, respectively. Then, the quantitative insulin sensitivity check index (QUICKI) was calculated, as indicated:

$$\:QUICKI=\:\frac{1}{\text{log}\left(fasting\:insulin\:[\mu\:U/ml]\right)+\text{l}\text{o}\text{g}\left(fasting\:glucose\:\right[mg/dl\left]\right)}$$

The results obtained were analyzed with Statistica 13.3 software (TIBCO Software Inc., Palo Alto, USA), using one-way analysis of variation (ANOVA) with the Dunnett test. The results obtained were presented as mean ± standard deviation. The results were considered statistically significant at a p value < 0.05 (* *p* < 0.05, ** *p* < 0.01). GraphPad Prism 9.0.0 software (Boston, USA) was used for visualization.

## Supplementary Information

Below is the link to the electronic supplementary material.

Supplementary Material 1

Supplementary Material 2

Supplementary Material 3

Supplementary Material 4

Supplementary Material 5

## Data Availability

The datasets used and/or analysed during the current study available from the corresponding author on request.
