# Supplementary material for: Ingested PET microplastics alter the metabolomic profile of the porcine pancreas
Source: Sci Rep. 2025 Nov 10;15:39227. doi: 10.1038/s41598-025-21915-5 (PMC12603213; doi:10.1038/s41598-025-21915-5)
Supplement: Supplementary file 5 — Supplementary Material 5 [file 41598_2025_21915_MOESM5_ESM.pdf]

Supplementary materials

Table S1 (a) List of peak intensities identified in the study after normalization against QC samples in negative ionization mode (ESI-) after treatment of the piglets with a low dose of PET microplastics. (b) List of statistically significantly different metabolites between control samples and those treated with a low dose of PET microplastics, identified in the study in negative ionization mode (ESI-). (c) KEGG pathways enrichment analysis of metabolites identified in the study in negative ionization mode (ESI-).

Table S2 (a) List of peak intensities identified in the study after normalization against QC samples in positive ionization mode (ESI+) after treatment of the piglets with a low dose of PET microplastics. (b) List of statistically significantly different metabolites between control samples and those treated with a low dose of PET microplastics, identified in the study in positive ionization mode (ESI+). (c) KEGG pathways enrichment analysis of metabolites identified in the study in positive ionization mode (ESI+).

Table S3 (a) List of peak intensities identified in the study after normalization against QC samples in negative ionization mode (ESI-) after treatment of the piglets with a high dose of PET microplastics. (b) List of statistically significantly different metabolites between control samples and those treated with a high dose of PET microplastics, identified in the study in negative ionization mode (ESI-). (c) KEGG pathways enrichment analysis of metabolites identified in the study in negative ionization mode (ESI-).

Table S4 (a) List of peak intensities identified in the study after normalization against QC samples in positive ionization mode (ESI+) after treatment of the piglets with a high dose of PET microplastics. (b) List of statistically significantly different metabolites between control samples and those treated with a high dose of PET microplastics, identified in the study in positive ionization mode (ESI+).
